# Dysregulation of KIF14 regulates the cell cycle and predicts poor prognosis in cervical cancer: a study based on integrated approaches

**DOI:** 10.1590/1414-431X2021e11363

**Published:** 2021-09-03

**Authors:** Li Xiao, Sisi Zhang, Qingyu Zheng, Shuirong Zhang

**Affiliations:** 1 Department of Obstetrics and Gynecology, Jingzhou Hospital, Yangtze University, Jinzhou, Hubei, China; 2 Department of Ultrasound, Zhijiang People's Hospital, Yichang, Hubei, China

**Keywords:** KIF14, Cervical cancer, Prognosis, Biomarker, Cell cycle

## Abstract

Cervical cancer (CC) is the most common malignant tumor in females. Although persistent high-risk human papillomavirus (HPV) infection is a leading factor that causes CC, few women with HPV infection develop CC. Therefore, many mechanisms remain to be explored, such as aberrant expression of oncogenes and tumor suppressor genes. To identify promising prognostic factors and interpret the relevant mechanisms of CC, the RNA sequencing profile of CC was downloaded from the Cancer Genome Atlas and the Gene Expression Omnibus databases. The GSE63514 dataset was analyzed, and differentially expressed genes (DEGs) were obtained by weighted coexpression network analysis and the edgeR package in R. Fifty-three shared genes were mainly enriched in nuclear chromosome segregation and DNA replication signaling pathways. Through a protein-protein interaction network and prognosis analysis, the kinesin family member 14 (KIF14) hub gene was extracted from the set of 53 shared genes, which was overexpressed and associated with poor overall survival (OS) and disease-free survival (DFS) of CC patients. Mechanistically, gene set enrichment analysis showed that KIF14 was mainly enriched in the glycolysis/gluconeogenesis signaling pathway and DNA replication signaling pathway, especially in the cell cycle signaling pathway. RT-PCR and the Human Protein Atlas database confirmed that these genes were significantly increased in CC samples. Therefore, our findings indicated the biological function of KIF14 in cervical cancer and provided new ideas for CC diagnosis and therapies.

## Introduction

Cervical cancer (CC) is the most common type of malignant tumor in females and has affected a vast number of women in the world, especially in sub-Saharan Africa and Southeastern Asia. Although the incidence and mortality of cervical cancer have declined due to vaccination, screening, and control of precancerous lesions, CC incidence is high in some less economically developed areas, affecting young women (peak 45-49) and advancing to the late stage (1). Approximately 570,000 new CC cases per year worldwide cause 31,000 deaths per year, making it the fourth leading cause of cancer death among women (2). Persistent high-risk human papillomavirus (HPV) infection is a leading factor that leads to cervical tumor occurrence (3); however, few women infected with HPV eventually develop cervical cancer. Other risk factors have been reported and include immunosuppression, smoking, pregnancy history, and long-term contraception, suggesting that the occurrence of CC is a multifactor, multistep complex process not only related to the environment but also involved in the aberrant expression of oncogenes and tumor suppressor genes (4). Therefore, it is necessary and urgent to identify sensitive and specific biomarkers that could predict CC prognosis and serve as a target for CC treatment.

In the past decade, high-throughput transcriptomics techniques (e.g., using microarrays and RNA sequencing) have been extensively used in the identification of cancer-related biomarkers, pathways, and drug targets (5). Several online databases, including the Gene Expression Omnibus (GEO) and The Cancer Genome Atlas (TCGA), are readily available, which allow transcriptomic, genomic, proteomic, and epigenomic data to be used for comprehensive analysis (6,7). Weighted gene coexpression network analysis (WGCNA) is a powerful genomic technique extensively used in the exploration of disease-related biomarkers (8). In the present study, we performed comprehensive bioinformatics analyses using the expression profiles of CC patients from the GEO database through WGCNA and differentially expressed gene (DEG) analysis. Subsequently, Kyoto Encyclopedia of Genes and Genomes (KEGG) pathway and Gene Ontology (GO) enrichment analyses were performed. Hub genes were detected by protein-protein interaction (PPI) network analysis and cytoHubba. The Kaplan-Meier curve of CC was drawn by the Gene Expression Profiling Interactive Analysis (GEPIA) database to identify the prognostic molecules in CC. Gene set enrichment analysis (GSEA) and correlation analysis were performed to further investigate the potential molecular mechanism and biological roles of potential hub genes.

## Material and Methods

### Data acquisition 

The expression profile of GSE63514 was downloaded from GEO (https://www.ncbi.nlm.nih.gov/geo/). GSE63514 contains 24 cervical normal tissues and 76 cervical intraepithelial neoplasia (CIN) tissues, which comprise 14 CIN1 lesions, 22 CIN2 lesions, 40 CIN3 lesions, and 28 CC tissues, which were analyzed with GeneChip RMA (GC-RMA). Later, the gene symbols were matched with probes after removing redundant data, and the “limma” package in R software 4.0 (https://bioconductor.org) was used to correct the background, normalize quantiles, and summarize quantiles.

### Construction of WGCNA and identification of modules

A weighted gene coexpression network was established using the “WGCNA” package in R and the one-step network construction and module detection function. First, the expression data were clustered, and obvious outliers were removed. Then, the soft-thresholding power of 7 was set according to the scale-free topology criterion. Furthermore, the average linkage hierarchical clustering dendrogram was used to explore gene modules based on a topological overlap matrix (TOM). A minModuleSize of 30 was selected to detect modules with different colors, and a mergeCutHeight of 0.25 was set to merge the similar modules automatically. Finally, the Spearman correlation coefficient was calculated to assess the correlation between the genes and clinical traits.

### Identification of differentially expressed genes (DEGs) in CC

The edgeR package in R (https://bioconductor.org) was applied to explore DEGs in CC. The criteria of false discovery rate (FDR) value <0.05 and |logFC| >1, upregulated genes (log2FC >1), and downregulated genes (log2FC ≤1) were set for significant DEGs based on the normalized gene expression levels.

### Screening of candidate genes

A Venn diagram program was performed to reflect the intersection between DEGs and red and green modules in WGCNA, which included 30 downregulated genes and 23 upregulated genes.

### Pathway enrichment analyses

To investigate the underlying mechanisms of shared DEGs, the Kyoto Encyclopedia of Genes and Genomes (KEGG) pathway analysis and Gene Ontology (GO), including gene molecular function, biological process, and cellular component, were performed using the “clusterProfiler” package in R (https://bioconductor.org).

### Construction of the protein-protein interaction (PPI) network

The interaction between proteins was analyzed through the Search Tool for the Retrieval of Interacting Genes (STRING) database (http://string-db.org, version 11.0) with a confidence score >0.41. PPI networks were established using the Cytoscape plugin cytoHubba (https://apps.cytoscape.org). The top 10 genes were selected as candidate hub genes based on the maximal clique centrality (MCC) algorithm.

### Gene set enrichment analysis

To explore the signaling pathways and biological characteristics associated with kinesin family member 14 (KIF14) expression in CC, the expression profile of CC from TCGA was used for KIF14 analysis through the GSEA software (http://www.gsea-msigdb.org). Pathways were selected based on the following criteria: normalized enrichment score >1 or ≤1, nominal P value <0.05, and FDR q-value <0.5.

### GEPIA database analysis

GEPIA (http://gepia2.cancer-pku.cn) is an online cancer database that provides fast and customizable functions based on TCGA and GTEx project data for comprehensive expression analysis by mining differentially expressed levels between cancer and noncancer patients (9). We explored the overall survival (OS) and disease-free survival (DFS) of patients with high and low KIF14 expression. Moreover, we investigated the correlation between the expression of KIF14 and markers of the cell cycle signaling pathway, including BUB1, TTK, PLK1, CREBBP, and CDK1.

### Cell culture and RT-qPCR analysis

Human cervical cancer cells (HeLa and SIHa) were purchased from the China Center for Type Culture Collection. Cells were maintained in DMEM (Servicebio, China) complete medium with 100 U/mL penicillin/streptomycin (YEASEN Biotech Co. Ltd., China) and 10% fetal bovine serum (FBS; YEASEN Biotech Co. Ltd.) at 37°C in a humidified atmosphere containing 5% CO_2_. Total RNA was extracted from the synovial membrane using TRIzol reagent, and cDNA synthesis was performed with a Hifair 1st Strand cDNA Synthesis kit (YEASEN Biotech Co., Ltd.) according to the manufacturer's protocol. Reverse transcription-quantitative polymerase chain reaction (RT-qPCR) was performed using Hieff qPCR SYBR Green Master Mix (YEASEN Biotech Co. Ltd.), and the gene expression levels were detected by an ABI 7500 Real-Time PCR system (Applied Biosystems; Thermo Fisher Scientific, Inc., USA). Relative mRNA expression was calculated with the 2^−ΔΔCT^ method compared to GAPDH expression. The primers are listed in Supplementary Table S1.

### Expression of KIF14 and cell cycle signaling pathways

The Human Protein Atlas (HPA, https://v15.proteinatlas.org/) (10) provides information on the tissue and cell distribution of all 24,000 human proteins, and it was used to validate the differential expression of KIF14 and the indexes of cell cycle signaling pathways at the protein level in the present study.

### Statistical analysis

Statistical analysis was carried out using GraphPad Prism 7.0 (GraphPad Software Inc., USA). Student's *t*-test was used for analyzing two groups with normal distribution. A P value of <0.05 was considered significant.

## Results

### Screening of DEGs among cervical normal, CIN, and CC tissues

**Figure 1:**
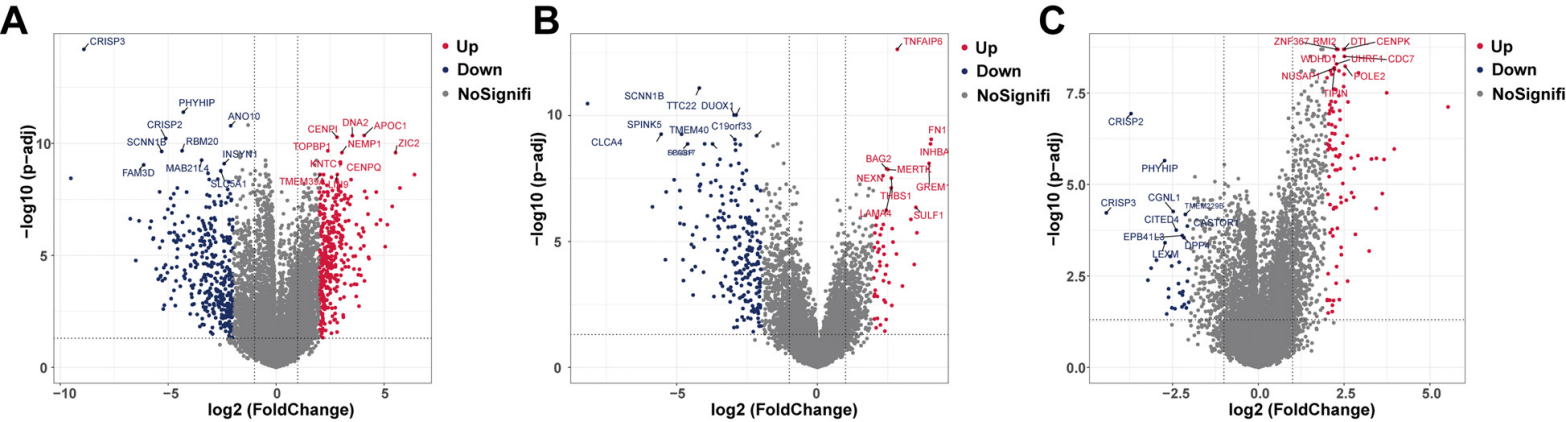
Screening of differentially expressed genes (DEGs) among cervical normal, cervical intraepithelial neoplasia (CIN), and cervical cancer (CC) tissues. **A**, DEGs between CC and normal tissue. **B**, between CC and CIN samples, and **C**, between CIN and normal tissue samples in the GSE63514 database are presented in volcano plots. The gray nodes represent genes that are not differentially expressed, and the blue and red dots represent downregulated and upregulated genes, respectively.

We used the microarray dataset (GSE63514) that included 128 cervical specimens, which were separated by histopathology into three disease stages: normal (24), CIN (80), and cancer (28). There were 2759 DEGs between CC and normal tissue samples, including 1741 upregulated genes and 1018 downregulated genes (Figure 1A). There were 1169 DEGs between CC and CIN samples, including 481 upregulated genes and 688 downregulated genes (Figure 1B). There were 2759 DEGs between CIN and normal tissue samples, including 609 upregulated genes and 218 downregulated genes (Figure 1C).

### Identification of significant gene modules

**Figure 2:**
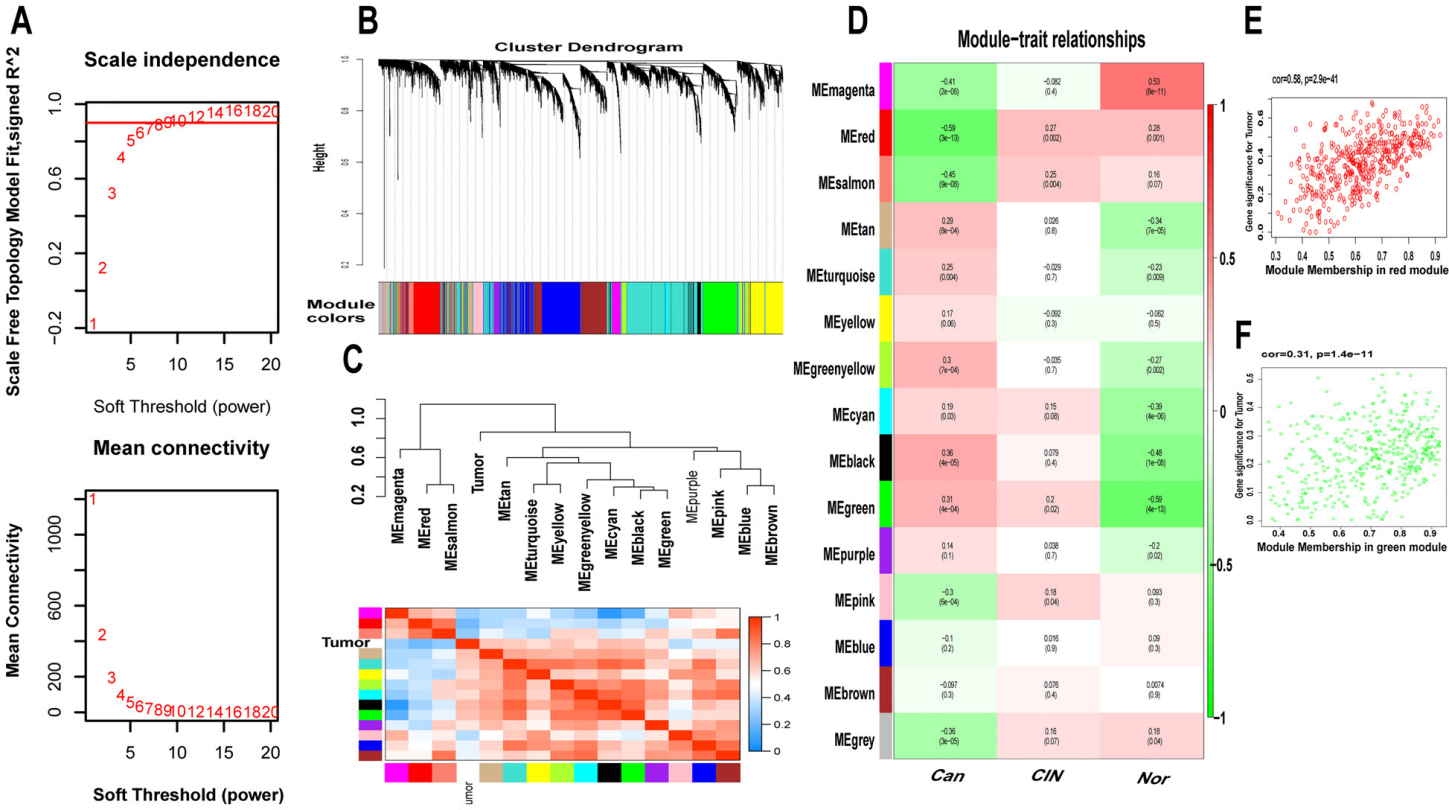
Identification of significant gene modules. **A**, A scale-free network was constructed in the GSE63514 database with the soft-thresholding power set at 7. **B**, Clustering dendrograms of genes based on dissimilarity topological overlap and module colors in the GSE63514 database are shown. **C**, A dendrogram is shown of consensus module eigengenes obtained by weighted coexpression network analysis on the consensus correlation and heatmap plot of the adjacencies of modules in the GSE63514 database. **D**, Scatter plots of gene significance relative to module membership in the GSE63514 database are shown. **E** and **F**, Scatter plots of red and green modules in the GSE63514 database are represented.

To further screen genes involved in the occurrence of CC, we constructed a WGCNA network based on the expression data of GSE63514. The soft-thresholding power was set at 7 with scale independence at 0.9 to ensure a scale-free network (Figure 2A). All 24,584 genes were assigned to 15 modules, among which 444 genes were assigned to the red module, and 455 genes were assigned to the green module (Figure 2B and C). Both modules were significantly related to clinical traits (red: correlation coefficient=−0.59, P<0.001; green: correlation coefficient=0.31, P<0.001; Figure 2D-F).

### Identification of coexpressed DEGs

**Figure 3:**
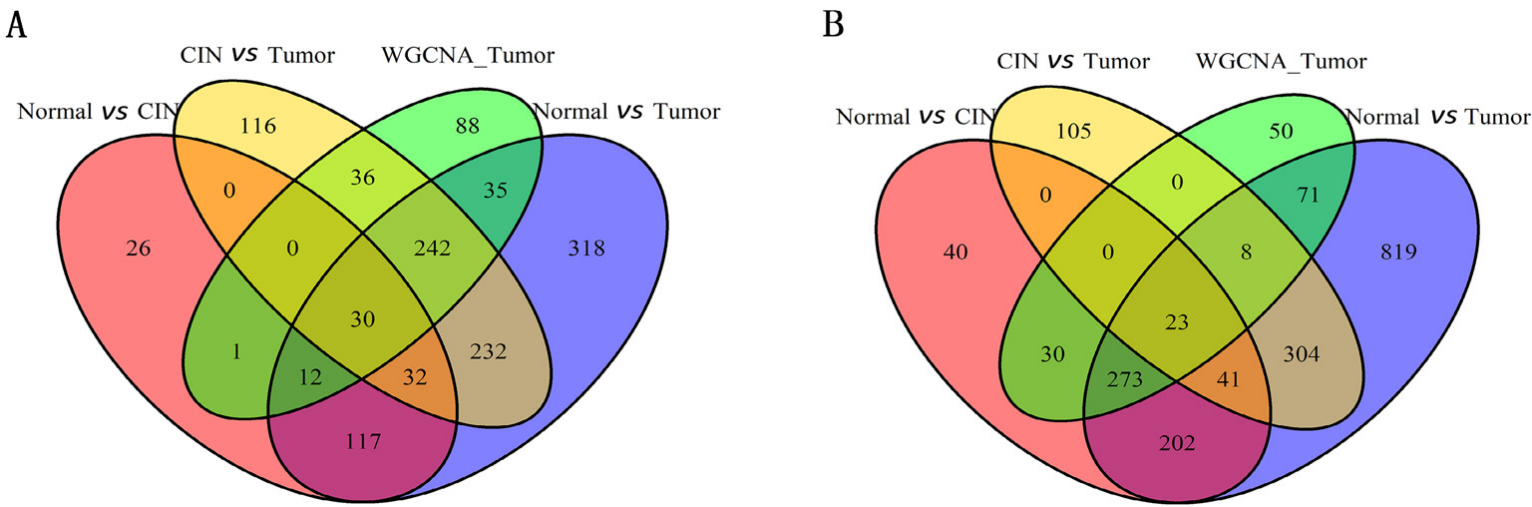
Identification of coexpressed differentially expressed genes (DEGs). **A** and **B**, Venn diagrams show downregulated and upregulated genes from both DEGs by weighted coexpression network analysis (WGCNA) and the edgeR package in R. Intersected areas represent the common DEGs in both databases. CIN: cervical intraepithelial neoplasia.

To further identify the key genes associated with cervical cancer, we compared the coexpressed genes in the red module with the downregulated DEGs between CC and normal tissue samples, between CC and CIN samples, and between CIN and normal tissue samples, and we compared the coexpressed genes in the green modules with the upregulated DEGs. Sets of 30 shared downregulated genes and 23 shared upregulated genes were obtained (Figure 3A and B).

### Functional annotation for coexpressed DEGs

**Figure 4:**
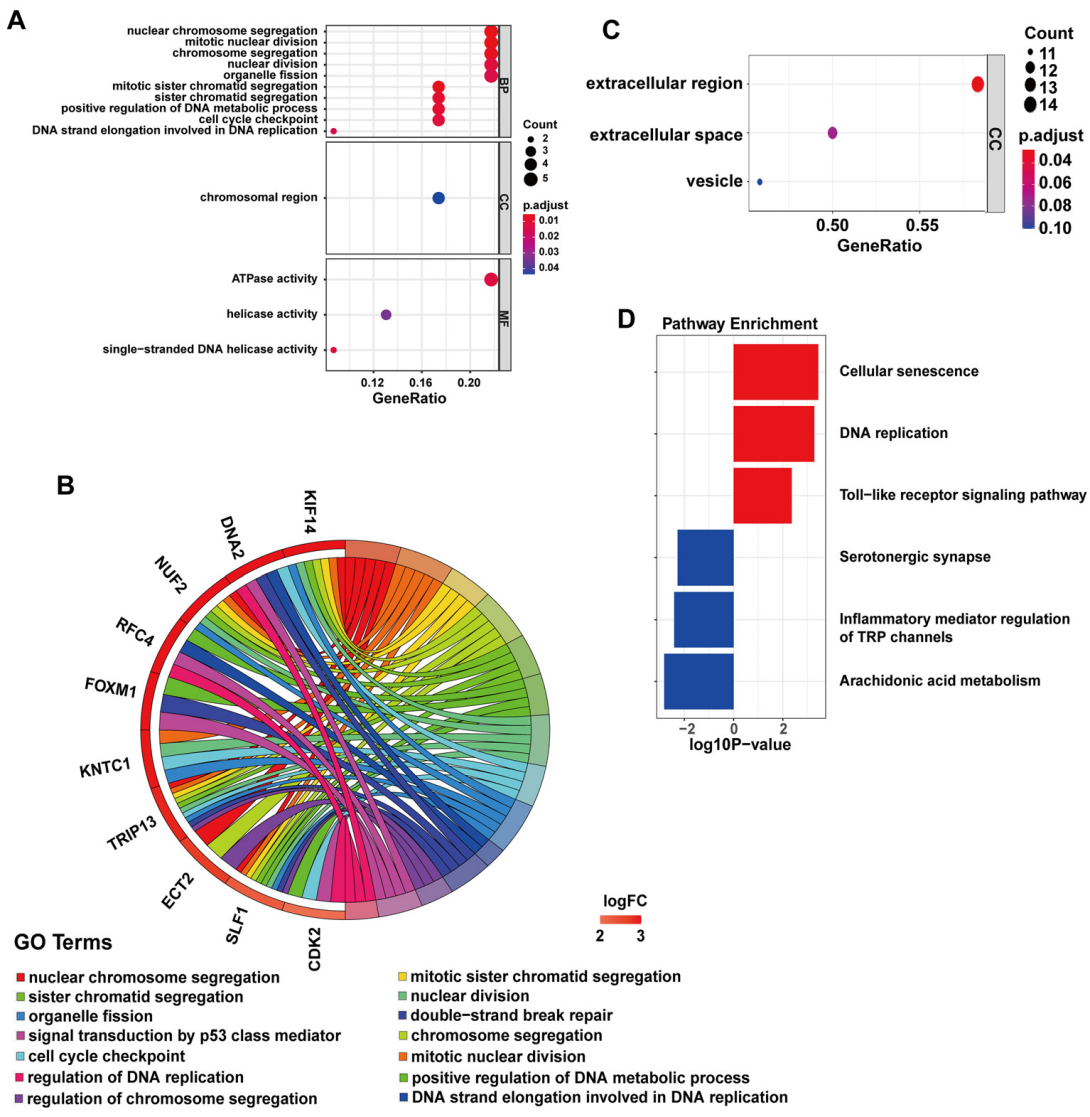
Functional annotation for coexpressed differentially expressed genes (DEGs). **A**, Gene Ontology (GO) analysis of the 23 shared upregulated genes, including biological process (BP), cellular component (CC), and molecular function (MF). **B**, Genes linked by ribbons to their enriched terms are presented in the GOChord plot. **C**, GO analysis results are shown of the 30 shared downregulated genes in the cellular component only (CC). **D**, KEGG pathway enrichment analysis results of the 53 shared genes are shown. P<0.05 (*t*-test).

Most of the upregulated DEGs were enriched in nuclear chromosome segregation, mitotic nuclear division, and chromosome segregation in biological processes. Some upregulated DEGs were enriched in the chromosomal region and ATPase activities in cell components and molecular functions (Figure 4A and B). Downregulated DEGs were enriched only in cellular components, mainly in extracellular regions (Figure 4C). The 23 upregulated DEGs were enriched in 3 KEGG pathways, including cellular senescence, DNA replication, and the Toll-like receptor signaling pathway. The significantly enriched downregulated DEGs involved 5-HT synapses, inflammatory regulation, TRP channel regulation, arachidonic acid metabolism, and other biological pathways (Figure 4D).

### Identification of hub genes

**Figure 5:**
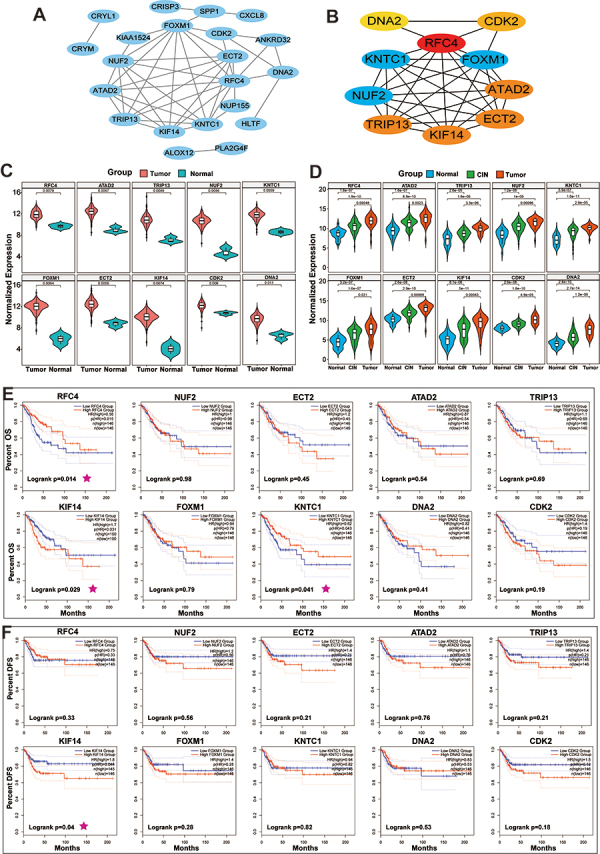
Identification of hub genes. **A**, Protein-protein interaction network analysis was performed. Twenty-one nodes and 45 edges were obtained with confidence scores ≥0.7. **B**, The top ten genes were selected as candidate hub genes according to the MCC algorithm. **C**, Violin plots present the expression of the ten hub genes in cervical cancer (CC) tissues compared to the cervical intraepithelial neoplasia (CIN) and cervical normal tissues in the GSE63514 database. **D**, Violin plots present the expression of the ten hub genes in CC tissues compared to cervical normal tissues in the TCGA database. **E**, Overall survival (OS) plots in response to the expression of the ten hub genes are shown. **F**, Disease-free survival (DFS) plots in response to the expression of the ten hub genes are shown.

String analysis was performed on 53 wild rabbit genes, and Cytoscape was used to construct a PPI network containing 21 nodes and 45 edges (Figure 5A). Then, the top ten genes were selected as potential hub genes based on the MCC algorithm through CytoHubba. Figure 5B shows that the 10 candidate hub genes were RFC4, ATAD2, TRIP13, NUF2, FOXM1, ECT2, KIF14, CDK2, KNTC1, and DNA2.

Furthermore, to investigate the credibility of the 10 candidate hub genes, we explored their expression levels in CC samples. In the GSE63514 database, the aforementioned genes were all overexpressed in CC tissues compared to CIN and cervical normal tissues (Figure 5C, P<0.05). In addition, we then downloaded the expression profile of CC from TCGA, and it was observed that the mRNA expression of these genes was also higher than that of cervical normal tissues (Figure 5D, P<0.05).

Finally, to determine the relationship between the expression of hub genes and DFS and OS in CC patients, Kaplan-Meier curves were generated using GEPIA. The results showed that the expression of three genes (KNTC1, KIF14, and RFC4) significantly correlated with the OS of CC patients (Figure 5E). There was a notably longer survival time in the patients with high expression of KNTC1 and RFC4 than in those with low expression; however, the two genes were overexpressed in CC (Figure 5C), which is contradictory to the prognosis. Only the patients with high expression of KIF14 displayed significantly shorter survival times than those with low expression. Furthermore, there was a significant negative correlation between the expression of KIF14 with the DFS of CC patients, while KNTC1 and RFC4 did not show similar results (Figure 5F). Thus, KIF14 may be a more important factor involved in the occurrence of CC, and we explored it further.

### Identification of KIF14-related signaling pathways by GSEA

**Figure 6:**
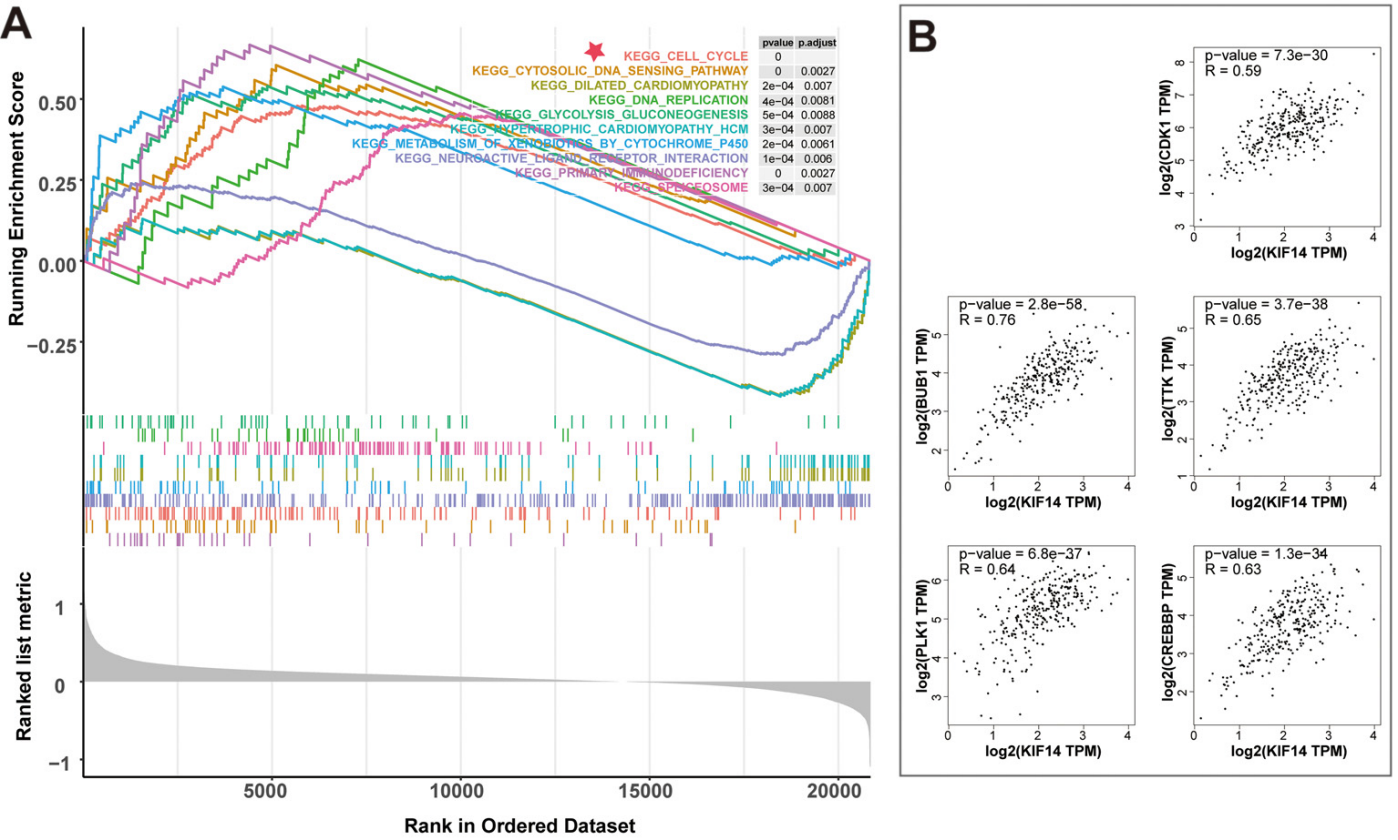
Identification of kinesin family member 14 (KIF14)-related signaling pathways by gene set enrichment analysis (GSEA). **A**, A merged enrichment plot is shown from GSEA including enrichment score, gene sets, and P values. Ten KIF14-related pathways are shown here. **B**, The correlation between KIF14 expression and the five indexes of the cell cycle, including BUB1, TTK, PLK1, CREBBP, and CDK1, were analyzed using Gene Expression Profiling Interactive Analysis (GEPIA).

To investigate differentially activated signaling pathways in CC, we conducted the GSEA between high- and low-KIF14-expression datasets from TCGA. Thirty-five significantly enriched signaling pathways were enriched based on the standard (p_adjusted_ <0.05 and FDR q values <0.05) (Supplementary Table S2). There were many well-known pathways including the cell cycle signaling pathway, the glycolysis/gluconeogenesis signaling pathway, and the DNA replication signaling pathway (Figure 6A).

The GSEA results and the GO enrichment analysis (Figure 6B) suggested that KIF14 was involved in regulating the cell cycle in CC, so we further explored the relationship between KIF14 and the cell cycle signaling pathway. Five indexes of the cell cycle, including BUB1, TTK, PLK1, CREBBP, and CDK1, were selected and identified as the important markers of the cell cycle signaling pathway. The correlation of KIF14 expression with the aforementioned genes was analyzed using GEPIA. Figure 6B shows a significant positive correlation between the expression of KIF14 with BUB1 (R=0.76), TTK (R=0.65), PLK1 (R=0.64), CREBBP (R=0.63), and CDK1 (R=0.59). Therefore, KIF14 may play an oncogenic role possibly through promoting cell cycle and cell proliferation in CC.

### Expression of KIF14 and indexes of cell cycle signaling pathways

**Figure 7:**
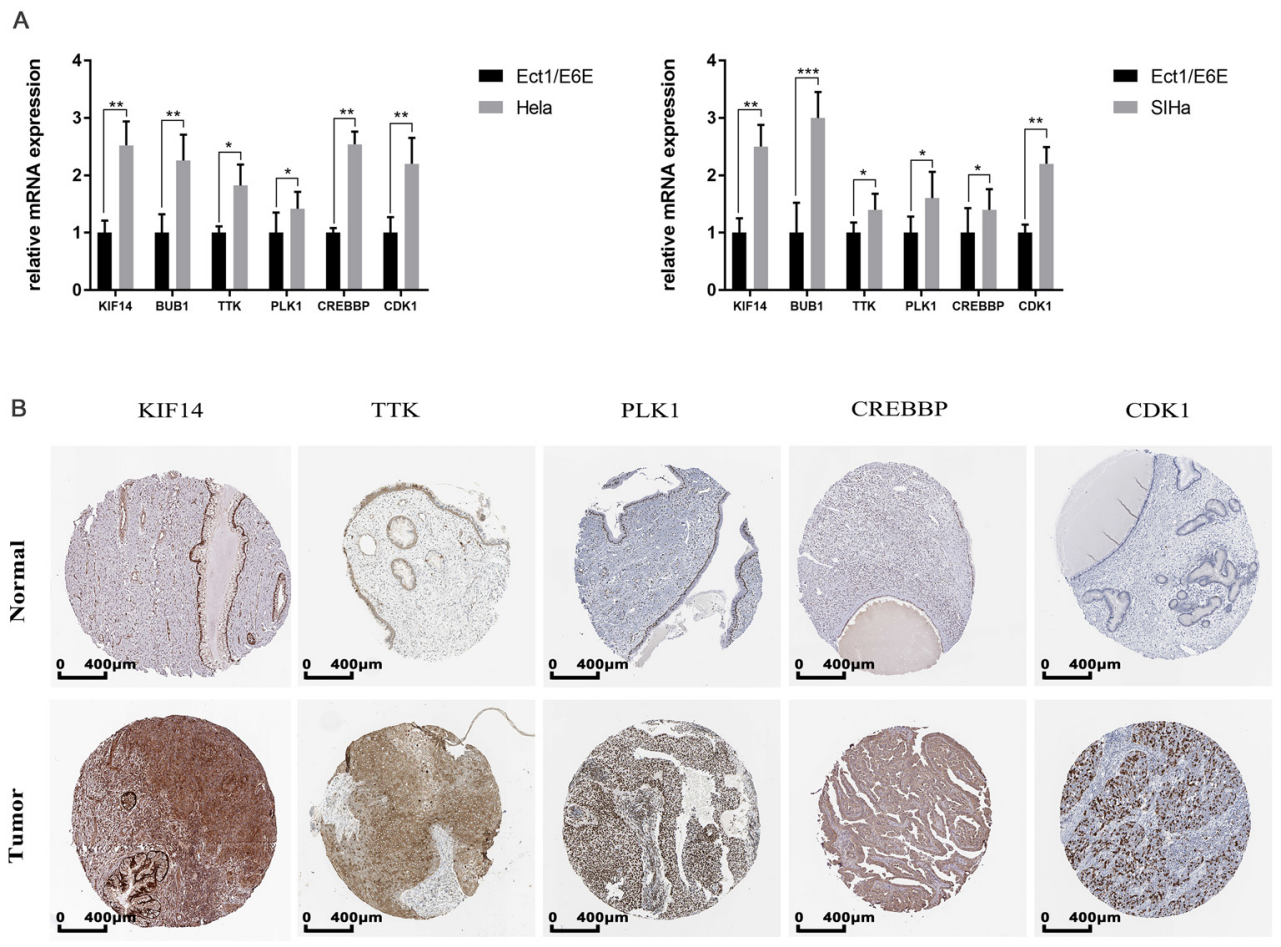
Kinesin family member 14 (KIF14) expression and the indexes of cell cycle signaling pathways. **A**, RT-PCR analysis results are shown of KIF14 and the indexes (BUB1, TTK, PLK1, CREBBP, and CDK1) of the cell cycle signaling pathways in human cervical cancer cells (HeLa and SIHa) and a normal cervical epithelial cell line (Ect1/E6E). **B**, The HPA database was utilized to analyze the protein expression of KIF14, TTK, PLK1, CREBBP, and CDK1 in normal and cervical cancer (CC) tissues. Scale bar, 400 μm. Data are reported as means±SD (n=3). *P<0.05; **P<0.01; ***P<0.001 (*t*-test).

To identify the expression of KIF14 and the indexes of cell cycle signaling pathways, we detected the expression of KIF14 and the above genes by RT-PCR. We found that human HeLa and SIHa CC cells overexpressed KIF14 and the other aforementioned genes, which were compared with the normal cervical epithelial cell line Ect1/E6E (Figure 7A). Furthermore, we observed that the protein expression of KIF14 was higher in CC tissues compared to normal cervix tissues based on the HPA database, and KIF14 was mainly localized in the cytoplasm and membrane (Figure 7B). Additionally, four of the five indexes of the cell cycle (TTK, PLK1, CREBBP, and CDK1) were included in the HPA, all of which were also highly expressed in CC (Figure 7B).

## Discussion

Our results suggested that shared upregulated DEGs played a more important role in CC pathogenesis compared with downregulated DEGs. Ten candidate hub genes were extracted through PPI network analysis of the set of 53 genes, and they were all upregulated in CC compared with CIN and normal cervical tissues. After OS and DFS analysis, only KIF14 was negatively associated with OS and DFS in CC patients. Therefore, KIF14 may be a critical oncogene involved in the onset and progression of CC, and it might also be useful as a diagnostic marker and specific therapeutic target for the ultimate management of CC.

KIF14, located on chromosome 1q32.1, contains a C-terminal motor domain, a citron kinase binding region, and an N-terminal extension for the binding of protein-regulating cytokinesis. The overexpression of KIF14 may lead to rapid and error-prone mitosis, which induces aneuploidy during tumorigenesis (11). Similar to our results, previous studies have demonstrated that KIF14 is overexpressed in some cancers, such as lung cancer, hepatocellular carcinoma, breast cancer, glioma, retinoblastoma, and ovarian cancer (12-15). More importantly, in aged transgenic mice and wild-type mice overexpressing KIF14, spontaneous tumor formation of fatal lymphomas was observed, which were mainly follicular and diffuse b-cell lymphomas (16), providing evidence that KIF14 may be an oncogene in the progression of multiple malignant tumors. In medulloblastoma, KIF14 overexpression at the protein level was notably associated with shorter progression-free survival and OS (17), and KIF14 downregulation significantly increased DFS and trended toward longer OS in lung cancer (18), which is consistent with our study that showed that KIF14 expression was an independent prognostic factor for the outcome of CC. Moreover, the upregulation of KIF14 promotes tumor proliferation and inhibits apoptosis, while KIF14 downregulation suppresses tumorigenicity *in vitro* and in xenografts (19). In addition, KIF14 has been reported to be a predictor of multiple cancer levels. Further, its downregulation changes the adhesion dynamics of lesions by increasing cell proliferation, thereby inhibiting cell migration and invasion (20-22). Nevertheless, the biological role of KIF14 and its underlying molecular mechanism in CC is still not clear.

To explore the mechanism of KIF14 in promoting CC onset, we analyzed the signaling pathway through GSEA and found that the cell cycle signaling pathway was closely related to KIF14, which was similar to the results reported for hepatocellular carcinoma, prostate cancer, and colorectal cancer (21,23,24). In colorectal cancer, Wang et al. (23) found that decreased expression of KIF14 leads to cell division failure and multinucleation, which inhibits cell cycle progression of nonviable daughter cells and hinders cell transition into S phase. Apart from interfering with cytokinesis, the fraction of cells in G2/M phase was significantly higher in glioma cell lines infected with KIF14-siRNA than in uninfected cells (U251 and U87 cells), indicating that KIF14 silencing induces G2/M phase arrest in glioma cells (25). BUB1, TTK, PLK1, CREBBP, and CDK1 are important cell cycle regulatory molecules and are commonly used as cell cycle markers (26). BUB1 plays a key role in accurately distributing chromosomes without mitotic spindle checkpoints and chromosome alignment, and TTK is a key element of the spindle assembly checkpoint (27). PLK1 plays a key role in mitosis by affecting chromosome separation, spindle assembly, and cytoplasmic division (28). Furthermore, PLK1 and TTK act cooperatively at the beginning of mitosis to establish a spindle assembly checkpoint by recruiting the Mad1:C-Mad2 complex to the kinetochores (29). Inhibiting CREBBP/EP300 bromodomain decreases GATA1- and MYC-driven transcription and causes the accumulation of cells in the G0/G1 phase of the cell cycle (30). CDK1 is an important regulator of many mitosis processes. As part of the cyclin A complex, CDK1 regulates the G2 phase and participates in G2/M transformation by forming the cyclin B complex (31). In addition, all aforementioned cell cycle markers are involved in tumorigenesis (32-36). Additionally, abnormal expression of KIF14 is often accompanied by abnormal expression of BUB1, TTK, PLK1, CREBBP, and CDK1 and affects the prognosis of several types of cancer (e.g., esophageal squamous cell carcinoma, glioblastoma, retinoblastoma, multiple myeloma, and acute myeloid leukemia) (37-39). Chen et al. (40) found the overexpressed genes (BUB1, BUB1B, TTK, and KIF14) in gastric cancer, which are important parameters included in the nomogram to predict the probability of relapse for gastric cancer patients. Therefore, our results showing that KIF14 was closely related to the expression of the aforementioned genes and played an important role in the pathogenesis of CC were consistent with these studies.

In summary, we have identified a novel predictor for the occurrence and prognosis of CC by integrating bioinformatics analysis. The KIF14 hub gene was overexpressed and related to the OS and DFS of CC patients. In addition, our results suggested that KIF14 was not only involved in the glycolysis/gluconeogenesis signaling pathway and the DNA replication signaling pathway but also tightly associated with the cell cycle signaling pathway and related to the expression of cell cycle markers in CC. Regrettably, we only performed a preliminary investigation on the function of KIF14 by analyzing TCGA and GEO data and have not confirmed the biological function of KIF14 through *in vivo* and *in vitro* experiments or determined how it modulates CC occurrence and development. Therefore, we will conduct systematic research on KIF14 to provide a theoretical basis for targeted CC therapy in the future. However, our current results revealed a biological role for KIF14 in cervical cancer pathogenesis and provided new ideas for CC diagnosis and therapy.

## Supplementary Material

Click here to view [pdf].
